# Next-generation sequencing library construction on a surface

**DOI:** 10.1186/s12864-018-4797-4

**Published:** 2018-05-30

**Authors:** Kuan Feng, Justin Costa, Jeremy S. Edwards

**Affiliations:** 1 Chemistry and Chemical Biology, University of New Mexico, Albuquerque, NM 87131 USA; 2 Special Projects, Centrillion Technologies, Palo Alto, CA 94303 USA; 3 Internal Medicine, Chemical and Biological Engineering, University of New Mexico, Albuquerque, NM 87131 USA; 4 University of New Mexico Comprehensive Cancer Center, Albuquerque, NM 87131 USA; 5 University of New Mexico Health Sciences Center, Albuquerque, NM 87131 USA

**Keywords:** Next generation sequencing, Transposases, Surface reaction

## Abstract

**Background:**

Next-generation sequencing (NGS) has revolutionized almost all fields of biology, agriculture and medicine, and is widely utilized to analyse genetic variation. Over the past decade, the NGS pipeline has been steadily improved, and the entire process is currently relatively straightforward. However, NGS instrumentation still requires upfront library preparation, which can be a laborious process, requiring significant hands-on time. Herein, we present a simple but robust approach to streamline library preparation by utilizing surface bound transposases to construct DNA libraries directly on a flowcell surface.

**Results:**

The surface bound transposases directly fragment genomic DNA while simultaneously attaching the library molecules to the flowcell. We sequenced and analysed a *Drosophila* genome library generated by this surface tagmentation approach, and we showed that our surface bound library quality was comparable to the quality of the library from a commercial kit. In addition to the time and cost savings, our approach does not require PCR amplification of the library, which eliminates potential problems associated with PCR duplicates.

**Conclusions:**

We described the first study to construct libraries directly on a flowcell. We believe our technique could be incorporated into the existing Illumina sequencing pipeline to simplify the workflow, reduce costs, and improve data quality.

## Background

The Human Genome Project is having a remarkable impact on the biomedical community, primarily due to the amazing reduction in sequencing costs, from $10 to less than $0.000001 per finished base in less than thirty years [1]. Exome sequencing is now routinely used in both research and clinical settings for the detection of inherited or acquired mutations related to disease, and the FDA has already listed over 100 drugs that have genotype information on their labels [2]. In addition, the use of whole genome sequencing is becoming more widespread. However, the simplicity of the Next-Generation Sequencing (NGS) pipeline could still be improved to further decrease the overall cost and increase the impact of NGS technology. Herein, we describe an approach to generate libraries directly on a flowcell surface, which will ultimately improve the efficiency of the NGS pipeline.

The traditional NGS library preparation protocol consists of three primary steps: fragmentation, adaptor ligation, and amplification. DNA molecules are first mechanically or enzymatically fragmented into 200~ 400 bp, and then sequencing adaptors are ligated to the fragments. Finally, after several cycles of PCR, the DNA library is ready to go through several quality control steps and load into the NGS instrument [3]. These steps typically take 8 to 10 h of hands-on work and expensive equipment is needed (e.g. Covaris). The Nextera kit improves this process by combining genome fragmentation and adaptor ligation into a single step, which is called tagmentation. Transposases used in the Nextera kit contain adaptors and when mixed with genomic DNA, they will shear the DNA and attach the adaptors to both ends of DNA fragments. This process is very efficient and only takes a few minutes. Though the library preparation has been simplified by using tagmentation, PCR is still required prior to loading the library into an NGS instrument. We believe that the NGS pipeline would be significantly improved if the library could be directly prepared on a flowcell surface, thus eliminating the need for upfront library construction. Ideally, genomic DNA could be directly loaded into the sequencing machine and no additional hands-on work. With our method, the NGS users could simply insert genomic DNA into a sequencing instrument and prepare a library directly on the flowcell surface.

Herein, we demonstrate that sequencing libraries can be successfully generated on flowcell surface with surface bound transposases. In this approach, DNA molecules are fragmented and linked to the flowcell surface by the Tn5 transposase. The linked DNA molecules are then ready for cluster generation. We believe our approach would simplify NGS pipeline significantly and contribute to the goal of sequencing a genome within $100.

## Results

### Overall process of tagmentation on polyacrylamide gel

**Fig. 1:**
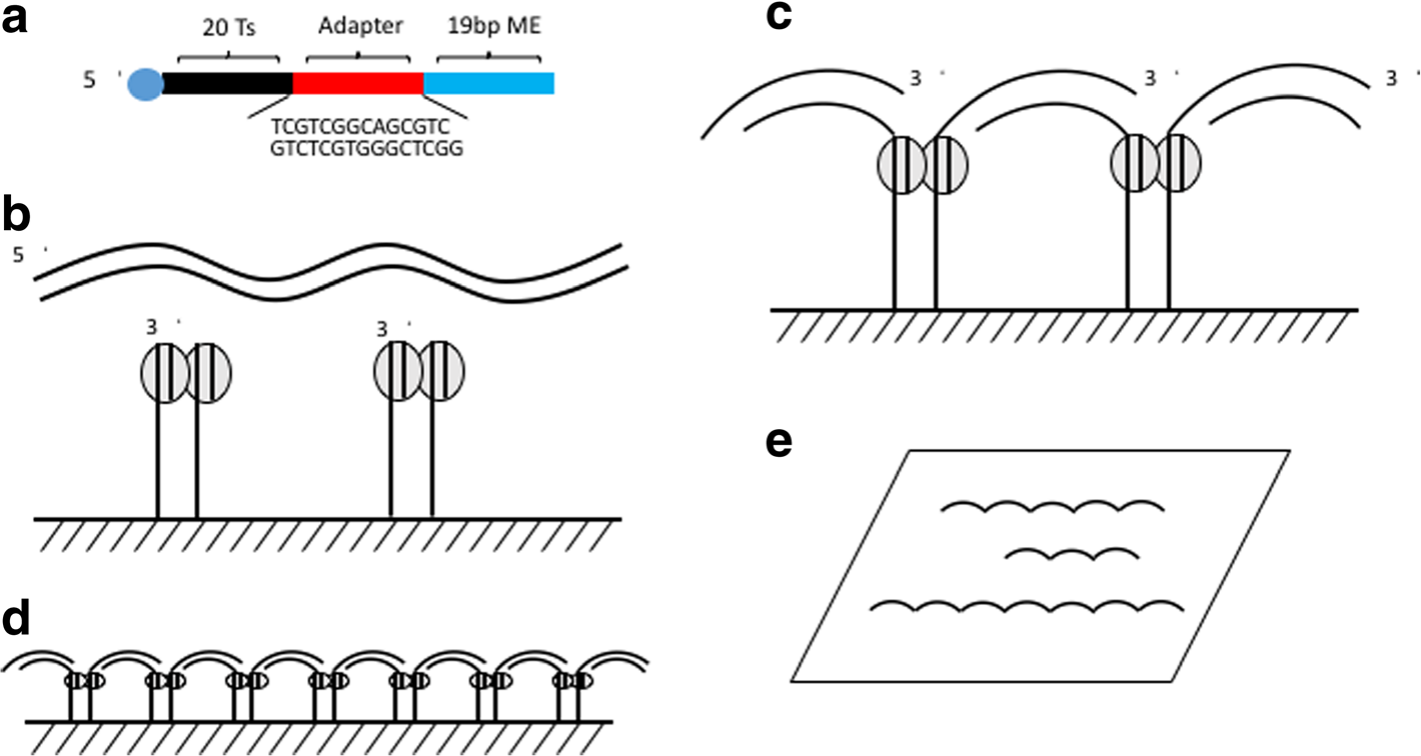
Tagmentation on surface. **a** oligonucleotides are attached to a ~ 10 μm thick polyacrylamide gel. The 5’ end of the oligos have an acrydite modification for attachment to the acrylamide matrix. **b** The Tn5 transposases are assembled on the dsDNA oligos on the polyacrylamide gel surface. **c** genomic DNA is linked to the surface through tagmentation via incubation at 55 °C for 20 min with TAPs buffer. **d**, **e** side view and vertical view of the linked DNA on surface

In order to perform the tagmentation on a solid surface rather than in solution, we first attached the Tn5 transposases on a surface. A thin (~ 10 μm) polyacrylamide hydrogel was used for the solid surface, as has been described previously for cluster generation [4–6], and the Illumina flowcell also has a very thin hydrogel layer on their flowcells [7]. We designed two oligonucleotides which contain the Illumina adapter sequences and a 19 bp mosaic end (ME) sequence, which the Tn5 will recognize. The 5′ end of the oligonucleotides was modified with an acrydite group to allow incorporation into the polyacrylamide gel as it polymerized (Fig. 1a), thereby linking the Tn5 transposases to the surface when the Tn5 binds the 19 bp ME (Fig. 1b). We also included a 20 base oligo dT spacer at the 5′ end of the oligonucleotides to make the ME more accessible to the transposases. Once the transposases were assembled on the polyacrylamide gel, genomic DNA and reaction buffer were applied to the surface for simultaneous fragmentation and attachment (or tagmentation) to the surface. The tagmentation reaction occurred at 55 °C, after which the DNA is fragmented and attached to the surface (Fig. 1c). In our experiments, the fragment size ranged from 200 bp to 1 kb. As shown in Fig. 1d and e, The DNA fragments are attached to the surface at both ends.

### Generating sequencing library with surface tagmentation

After the tagmentation one could directly perform cluster generation on the surface, as previously described in the literature [8–10]. However, herein, to confirm the library that we generated, following tagmentation we performed a polymerase extension step to fill the 9 bp gap created by Tn5 [11], as illustrated in Fig. 1c. To evaluate the quality of the surface bound library molecules, we transferred the hydrogel to a standard 200 μl PCR tube and recovered the library molecules by PCR. Finally, we sequenced the library on a MiSeq sequencer.

**Fig. 2:**
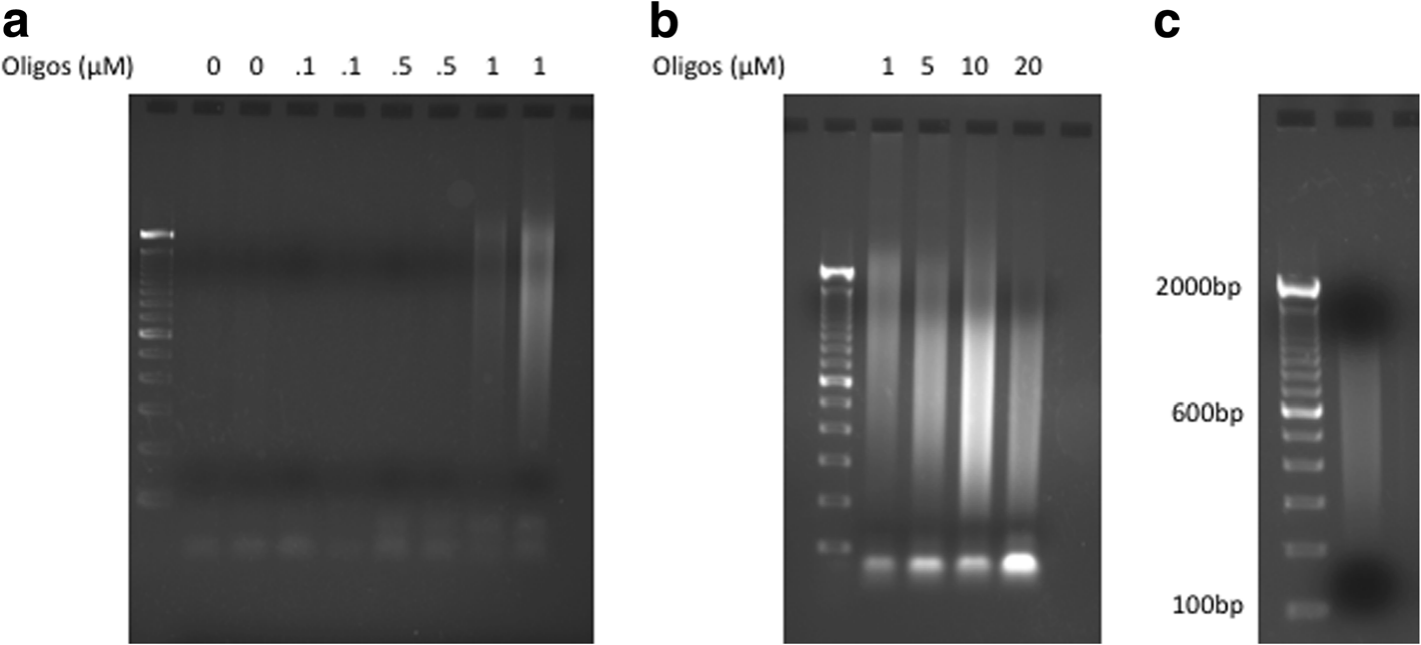
*Drosophila* sequencing library. **a** and **b** Agarose gel (2%) demonstrating the PCR products from surface tagmentation, various amount of acrydite oligonucleotides were used when casting the poly-acrylamide gel. **c** Agarose gel (2%) demonstrating the sequencing library after surface tagmentation and 16 cycles of PCR. The sequencing library was size selected with Agencourt Ampure XP beads

One would expect that the library quantity and the size of the fragments would be related to the density of immobilized oligonucleotides, which controls the density of immobilized transposases on the surface. Therefore, we generated sequencing libraries on multiple surfaces with oligonucleotide concentrations of 0.1 μM, 0.5 μM, 1 μM, 5 μM, 10 μM, and 20 μM (Fig. 2a and b), and the library molecules shifted to smaller sizes when we increased the oligonucleotides concentration (Fig. 2b). We were not able to generate a sequencing library with oligonucleotide concentrations below 1 μM (Fig. 2a). Finally, we sequenced a *Drosophila* genomic DNA library generated on a surface with an oligonucleotide concentration of 1 μM. As shown in Fig. 2c, the fragments size ranges from 200 bp to around 1 kb for this library, which is adequate for sequencing on an Illumina MiSeq.

**Fig. 3:**
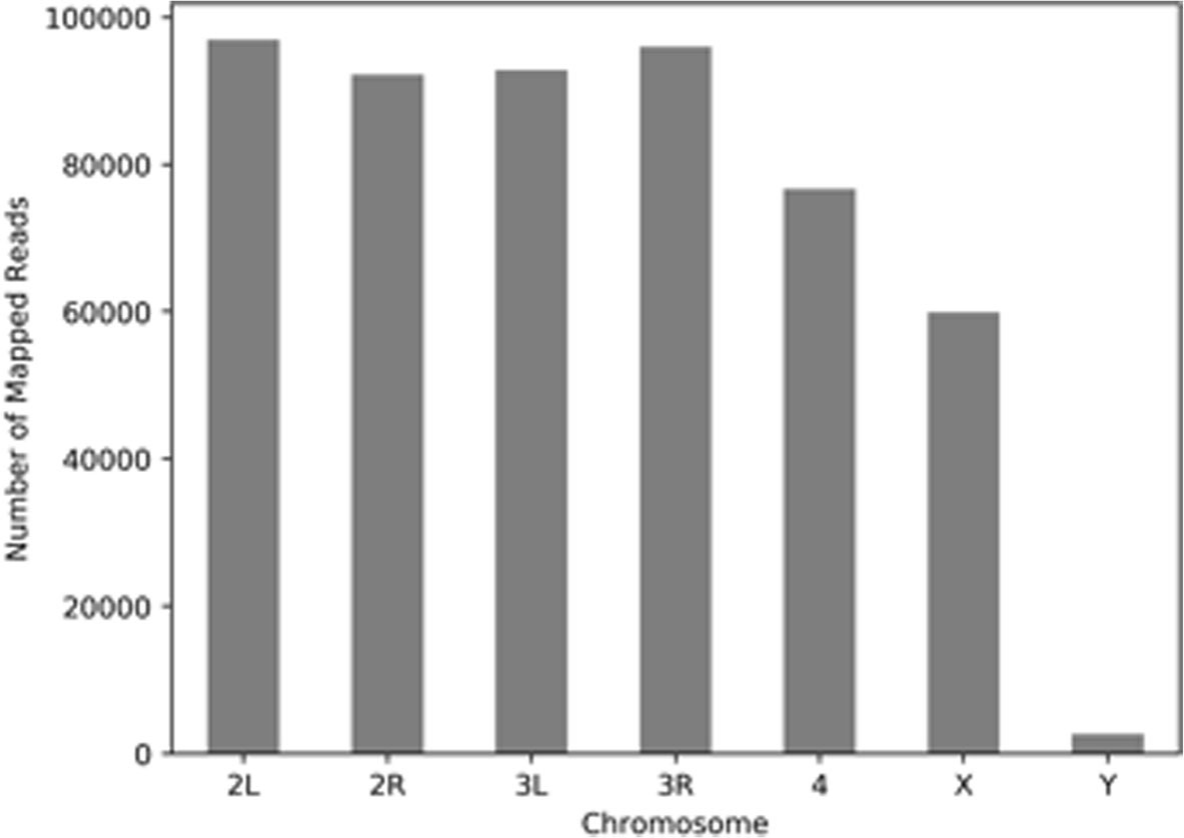
Sequencing reads distribution. All reads were mapped to the reference genome with BWA while only reads with mapping quality higher than 30 were used. Reads count was normalized to chromosome length

After sequencing, the resulting data was mapped to the reference genome [12] with BWA [13]. 97.3% of the reads were aligned to the reference genome with 1.8% PCR duplicates. The reads mapped uniformly across the *Drosophila* genome (Fig. 3). To further address sequencing bias, we downsampled the data to 1.2× depth of coverage and compared the actual breadth of coverage to the theoretical expected value (Table 1). We then compared our genome sequencing library to a library made from a commercial Illumina Nextera Kit, which used an in solution tagmentation technique (Table 1). When the two libraries were analysed at a similar sequencing depth, the coverage was comparable, with the surface bound library being slightly better (58.5% vs. 52.0%). Although the expected coverage at this sequencing depth should be around 71%.

**Table 1:** Comparison NGS libraries made by surface tagmentation and Nextera kit. Nextera data was downloaded from NCBI Sequence Read Archive (SRA, ERR481289). Reads from our surface tagmentation and the Nextera data were downsampled with Picardtools (PROBABILITY = 0.5) to have approximately 1.2× coverage. The expect breadth of coverage was calculated according to the formula Cb = 1–1/e^Cd^, where Cd stands for the depth of coverage while Cb stands for the breadth of coverage

Method	Depth of Coverage	Breadth of Coverage	Expect Breadth of Coverage
Surface tagmentation	1.249	58.5%	71.3%
Nextera Kit	1.272	52.0%	72.0%

### Surface tagmentation with combed DNA

**Fig. 4:**
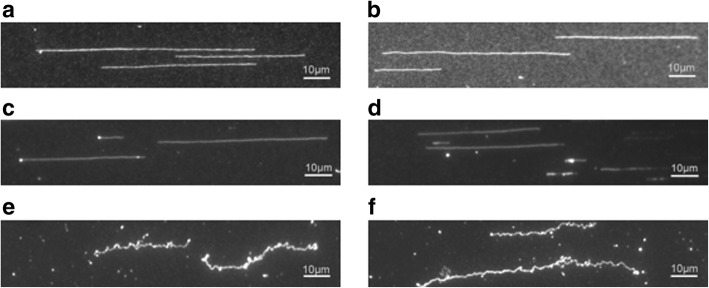
Combed DNA on a surface. **a** and **b** YoYo-1 stained DNA stretched on PDMS. **c** and **d** Combed DNA on poly-acrylamide gel before tagmentation. **e** and **f** Combed DNA on poly-acrylamide gel after tagmentation

Tagmentation has several advantages over other NGS library preparation methods, and requires less starting material. As little as 50 ng of DNA is enough to generate a library using Tn5 tagmentation in solution [14]. However, our approach requires 300 ng since the surface bound Tn5 reduces the DNA collision frequency. In order to solve this potential problem, we used combed DNA on a surface rather than DNA in solution. DNA combing is a technique to stretch DNA on a hydrophobic surface by a receding air-water meniscus [15]. We first combed DNA on Polydimethylsiloxane (PDMS) and then transferred to a polyacrylamide surface (Fig. 4a, b, c and d). In this way, DNA molecules are more likely to be captured by the transposases. Therefore, we can use a much lower concentration of DNA. Another benefit for using combed DNA is that DNA molecules will be kept in their original shape with minimal shearing. As can be visualized in Fig. 4, these DNA fibers can be as long as 100 μm, which corresponds to approximately 200 kb. Once combed DNAs were transferred to the polyacrylamide gel, they were tagmented by the transposases and linked to the surface (Fig. 4e and f). By using combed DNA, we could generate a library with as little as 50 ng of DNA.

## Discussion

Library preparation is the first step in the NGS pipeline, and the process has been standardised and several kits are commercially available. With these kits, a standard sequencing library can be prepared in around 10 h. The Nextera kit from Illumina, which utilizes Tn5 transposases to generate the sequencing library, dramatically reduces the hands-on time to 2 h by tagmentation [16], and this kit can generate a reasonable library for NGS. However, the kit itself is expensive and it still requires the sequencing library to be produced prior to injection to the sequencing instrument.

In order to make this technique more accessible to small labs, Picelli and colleagues cloned a Tn5 transposase [17], which generates comparable sequencing libraries to the Nextera kit. Using this enzyme, we have developed a method to generate DNA libraries directly on a flowcell surface, and ultimately within the sequencing instrument. In our approach, the Tn5 transposases are first attached to a polyacrylamide gel surface, which then fragments genomic DNA molecules and simultaneously links them to the surface. Once the DNA molecules are linked to the surface, one could generate clusters on the same surface and directly sequence the clusters [18], This approach could significantly simplify the whole library preparation and sequencing procedure. However, in this study to confirm the quality of the libraries generated by surface tagmentation, we constructed a *Drosophila* genome library by surface tagmentation and then extracted the library for sequencing to compare the overall performance to solution-based tagmentation.

Generally, the NGS library preparation requires 100 ng ~ 1 μg of genomic DNA to start with while the Nextera kit only use 50 ng. While with our method, 300 ng of genomic DNA was used. It is acceptable but not ideal if the DNA material is precious or difficult to acquire. To overcome this shortcoming, we made use of the DNA combing strategy and successfully reduced the starting material to 50 ng. DNA molecules were first combed on PDMS and then transferred to a flowcell surface. We made the PDMS surface in advance, and it only took several minutes to transfer the DNA from the PDMS to the flowcell surface. Ideally, we could comb DNA directly on flowcell surface, however, the DNA combing process was not as efficient on an acrylamide surface since it is hydrophilic. Two possible solution would be replacing the polyacrylamide gel with another hydrophobic surface or optimizing the combing conditions, i.e. pH or salt concentration [19].

Overall, our surface tagmentation strategy produced sequencing results that are comparable to those prepared in solution, while significantly simplifying the pre-sequencing library construction procedure. With DNA fragmented and attached to the flowcell surface, the DNA molecules are ready for cluster generation with no additional PCR amplification. To go one step further, the surface tagmentation step could be automated in a sequencing instrument, and the original DNA material can be loaded directly onto a sequencing flowcell without any hands-on work.

## Conclusion

In this study, we developed an approach to generate DNA libraries directly on a flowcell surface. With Tn5 transposase, we successfully generated a *Drosophila* sequencing library by surface tagmentation and the performance was comparable to the Nextera kit. Ultimately, our approach would allow cluster generation right after the tagmentation without any PCR, which drastically simplifies the whole NGS pipeline.

## Methods

### Genomic DNA isolation

Genomic DNA was extracted from *Drosophila melanogaster* (female) using phenol chloroform. DNA was quantified with Qubit dsDNA BR Assay Kit (Life Technologies) and stored at − 20 °C. pTXB1-Tn5 plasmid was acquired from Addgene (plasmid#60240).

### Preparation of poly-acrylamide gel surface

Clean glass slides were salinized with bind-silane (GE Healthcare) overnight at room temperature. 5 μl of poly-acrylamide gel mix (4% acrylamide/bis, 1 μM pre-annealed acrydite modified double-stranded oligonucleotides (equal molar of Tn5A/Tn5R and Tn5B/Tn5R), 0.005% TEMED and 0.005% ammonium persulfate) was loaded between the salinized slide and a 22 × 22 mm coverslip. The acrylamide polymerized for 1 h at room temperature. To wash off the excess acrylamide monomer and unbounded primers, the slide was incubated in 40 ml of 0.5× SSC on a shaker for 30 min. The acrydite modified oligonucleotides sequences are as follows: Tn5A, 5’ - [Arcydite] TTTTTTTTTTTTTTTTTTTTTCGTCGGCAGCGTCAGATGTGTATAAGAGACAG – 3’; Tn5B, 5’ - [Arcydite]TTTTTTTTTTTTTTTTTTTTTGTCTCGTGGGCTCGGAGATGTGTATAAGAGACAG – 3’; Tn5R, 5’ - [phos]CTGTCTCTTATACACATCT – 3’.

### DNA combing

Genomic DNA was stained with YoYo-1 (ThermoFisher) in phosphate buffered saline (pH 8.3) overnight at 4 °C. Polydimethylsiloxane (PDMS) coated coverslips were immersed into a reservoir containing YoYo stained DNA and incubated for 1 h at room temperature. PDMS coated coverslip was pulled up and out of the reservoir at 300 μm/s. Stretched DNA was visualized using an inverse fluorescence microscope (Keyence).

### Surface tagmentation and library preparation

Tn5 transposases, purified as previously described [17], were loaded onto the poly-acrylamide gel slide and incubated at 37 °C for 1 h. The slide was then washed several times with Tn5 wash buffer (100 mM Tris-HCl, 200 mM NaCl, 1 mM EDTA and 0.2% Triton-X100), and the surface was allowed to dry while preparing the tagmentation mix (1 μl 10× TAPS-MgCl_2_, 2 μl 40% PEG8000, 4 μl H_2_O and 3 μl 200 ng/μl DNA). When using combed DNA, they were transferred from PDMS by pressing the PDMS onto the Tn5 transposases preloaded polyacrylamide gel surface for 5 min. Tagmentation and library PCR were performed as previously described [17]. Briefly, the tagmentation was performed at 55 °C for 20 min and followed by several washes with nuclease free water. To confirm the library, the poly-acrylamide gel was then scraped off the slide and transferred to a 0.2 ml tube for library PCR using standard *Taq* polymerase (To make a 50 μl reaction: 5 μl of 10× standard buffer, 0.4 μM primer mix, 0.25 U *Taq* polymerase and nuclease free water). The PCR performed as: 68 °C for 3 min, 95 °C for 30 s, then 16 cycles of 95 °C for 15 s, 63 °C for 30 s and 68 °C for 3 min. PCR primers sequences are as follows: forward primer: 5’- AATGATACGGCGACCACCGAGATCTACACTCGTCGGCAGCGTC-3’; reverse primer: 5’-CAAGCAGAAGACGGCATACGAGATGTCTCGTGGGCTCGG-3’. The PCR products were purified and size selected by Agencourt AMPure XP beads (Beckman Coulter). The library was quantified by qPCR.

### MiSeq sequencing

The library was diluted to final concentration of 2 nM and 5% PhiX was spiked in as control. Sequencing was performed on a MiSeq instrument with the Nano Kit V2 in paired 150 bp mode (Illumina).

### Data analysis

All sequencing reads were mapped to the reference genome using BWA [13]. The resulting bam file was sorted and indexed using samtools [20]. Reads with mapping quality lower than 30 were removed. PCR duplicates were dumped using Picardtools. Coverage analysis was performed by homemade shell scripts. Nextera sequencing data was downloaded from NCBI Sequence Read Archive (SRA; http://www.ncbi.nlm.nih.gov/sra) under accession number ERR481289.

## Abbreviation

PDMS: Polydimethylsiloxane
